# Of the importance of a leaf: the ethnobotany of *sarma* in Turkey and the Balkans

**DOI:** 10.1186/s13002-015-0002-x

**Published:** 2015-04-03

**Authors:** Yunus Dogan, Anely Nedelcheva, Łukasz Łuczaj, Constantin Drăgulescu, Gjoshe Stefkov, Aida Maglajlić, Jonathan Ferrier, Nora Papp, Avni Hajdari, Behxhet Mustafa, Zora Dajić-Stevanović, Andrea Pieroni

**Affiliations:** Buca Faculty of Education, Dokuz Eylul University, 35150 Buca, Izmir Turkey; Department of Botany, University of Sofia, Blvd. Dragan Tzankov, 1164 Sofia, Bulgaria; Department of Botany, Institute of Applied Biotechnology and Basic Sciences, University of Rzeszów, Werynia 502, 36-100 Kolbuszowa, Poland; Department of Ecology and Environmental Protection, Lucian Blaga University, Dr. Ioan Raţiu St. 5–7, Sibiu Romania; Department for Pharmacognosy, Phytochemistry and Pharmaceutical Botany, Faculty of Pharmacy, University Ss. Cyril and Methodius, Skopje, Macedonia; Via Bertolina 79, I-12084 Mondovì (Cuneo), Italy; Department of Cellular and Molecular Medicine, University of Ottawa, Ottawa, K1H 8 M5 Canada; Ottawa Hospital Research Institute, Chronic Disease Program, Ottawa, K1Y 4E9 Canada; Department of Pharmacognosy, University of Pécs, Rókus 2, 7624 Pécs, Hungary; Institute for Biological and Environmental Research, University of Prishtina “Hasan Prishtina”, Mother Teresa Str., 10 000 Prishtinë, Republic of Kosovo; Department of Agrobotany, Faculty of Agriculture, University of Belgrade, Nemanjina 6, 11080 Zemun, Belgrade, Serbia; University of Gastronomic Sciences, Piazza Vittorio Emanuele 9, 12060 Bra/Pollenzo, Italy

**Keywords:** Ethnobotany, Balkans, Turkey, *Sarma*, Gastronomy

## Abstract

**Background:**

*Sarma* - cooked leaves rolled around a filling made from rice and/or minced meat, possibly vegetables and seasoning plants – represents one of the most widespread feasting dishes of the Middle Eastern and South-Eastern European cuisines. Although cabbage and grape vine *sarma* is well-known worldwide, the use of alternative plant leaves remains largely unexplored. The aim of this research was to document all of the botanical taxa whose leaves are used for preparing sarma in the folk cuisines of Turkey and the Balkans.

**Methods:**

Field studies were conducted during broader ethnobotanical surveys, as well as during ad-hoc investigations between the years 2011 and 2014 that included diverse rural communities in Croatia, Bosnia and Herzegovina, Serbia, Kosovo, Albania, Macedonia, Bulgaria, Romania, and Turkey. Primary ethnobotanical and folkloric literatures in each country were also considered.

**Results:**

Eighty-seven botanical taxa, mainly wild, belonging to 50 genera and 27 families, were found to represent the bio-cultural heritage of *sarma* in Turkey and the Balkans. The greatest plant biodiversity in *sarma* was found in Turkey and, to less extent, in Bulgaria and Romania.

The most commonly used leaves for preparing sarma were those of cabbage (both fresh and lacto-fermented), grape vine, beet, dock, sorrel, horseradish, lime tree, bean, and spinach. In a few cases, the leaves of endemic species (*Centaurea haradjianii*, *Rumex gracilescens*, and *R. olympicus* in Turkey) were recorded.

Other uncommon sarma preparations were based on lightly toxic taxa, such as potato leaves in NE Albania, leaves of *Arum*, *Convolvulus*, and *Smilax* species in Turkey, of *Phytolacca americana* in Macedonia, and of *Tussilago farfara* in diverse countries. Moreover, the use of leaves of the introduced species *Reynoutria japonica* in Romania, *Colocasia esculenta* in Turkey, and *Phytolacca americana* in Macedonia shows the dynamic nature of folk cuisines.

**Conclusion:**

The rich ethnobotanical diversity of *sarma* confirms the urgent need to record folk culinary plant knowledge. The results presented here can be implemented into initiatives aimed at re-evaluating folk cuisines and niche food markets based on local neglected ingredients, and possibly also to foster trajectories of the avant-garde cuisines inspired by ethnobotanical knowledge.

## Introduction

Turkey and the Balkans currently represent two exemplar arenas for ethnobiologists, since these regions can be considered both biological and cultural hotspots. Herein, the Balkans are defined as the South-Eastern European territory located south of the Danube-Sava-Kupa river systems line (i.e., the territory that includes the countries of Bosnia and Herzegovina, Serbia, Kosovo, Montenegro, Albania, Bulgaria, Macedonia, Greece, as well as the European part of Turkey, a small portion of Romania, and most of Croatia). In the last decade, Turkey and the Balkans have become popular field research locations aimed at documenting traditional environmental knowledge (TEK). Much of this recent research has focused on exploring this region’s uncommon, extremely rich, bio-cultural heritage, and also on valorizing local folk knowledge systems into sustainable rural projects that might improve the holistic well-being of the local communities, which in a significant portion of the Western Balkans have been recently heavily affected by the most recent Yugoslavian Wars [1].

Most of such studies have focused on folk knowledge related to plants, but some have also investigated the animals-humans nexus [2-4]. Among the ethnobotanical and environmental-anthropological studies published in international journals within the past decade and indexed in important scientific databases (i.e., PubMed, Scopus, WoK), only a few have also analyzed the wild food plant and mushrooms knowledge of the local communities in the Balkans and Turkey. Wild food and mushroom knowledge has been recorded in use among coastal, rural, and isolated mountainous communities in Bosnia [5-7], Bulgaria [8], Macedonia [9,10], Greece [11], Albania [12-16], in the Balkan portions of Croatia [17-19] and Romania [20], as well as in diverse areas of Turkey [21-26]. Moreover, a recent book focusing on Balkan ethnobiology included contributions on the consumption of wild food plants [1].

The term *sarma*, meaning “wrapped” in Turkish, defines leaves (raw or more often shortly blanched, or kept in salt brine) rolled around a filling made of rice, bulgur and, or minced meat, possibly vegetables and seasoning plants (especially onion), and gently cooked (stewed or boiled) in a pot and generally consumed warm (with meat) or cold (without meat).

*Sarma* represents a pillar of the traditional cuisines of the former Ottoman territories: Turkey, Persia, the Balkans, the Middle East, and Northern Africa. In some of these contexts, the overarching term *dolma* is also sometimes used, especially for grape vine leaf-based *sarma*, although it would be maybe more correct to only apply this term to stuffed vegetables (tomatoes, egg plants, peppers, onions, potatoes, artichoke, zucchini), in which the filling is not completely wrapped or covered by plant tissues.

*Sarma* has long-represented (at least for four centuries) a crucial festivity dish in many areas of the Ottoman Empire and was present on the menus of palaces and official residences (*konaks*), where it was prepared as a main course or as a side dish to a main course meat meal [27].

Since the origin of all Turkic populations is rooted in the Central Asiatic pastoralism, generally characterized by a large consumption of meat and dairy products and a low consumption of vegetables, gastronomy historians agree that *sarma* preparations may have possibly developed after Turks settled in Anatolia, where their diet was enriched by a large number of cultivated vegetables [26], whereas the Ottoman cuisine is surely the result of a complex *metissage* and interactions between the Turkish cuisine and a number of other cuisines native to the surrounding territories (notably the Arabic, Persian, the Mediterranean, and East European cuisines) [28-32].

According to the German traveller and merchant Hans Dernschwam, who visited Istanbul between 1552 and 1555, *dolma* and *sarma* were then commonly consumed, and fresh grape vine leaves were sold in many places for preparing *sarma.* Dernschwam’s diaries note that the filling of *sarma* was made from meat and that *sarma* was cooked together with unripe, sour plums [33]. Other historical sources testify that in 1640 cabbage *sarma* was sold in Istanbul, while in 1660 cabbage *sarma* was on the menu of some dinner parties of wealthy men [26].

Turkish cookbooks written in the 19th Century underline the importance of a balance between the sour and sweet tastes in *sarma* [34], a principle that was probably borrowed from the Persian cuisine. In addition to the inclusion of minced onions occurring in the filling, the wrapped leaves were cooked adding lemon, and sometimes also unripe plums, sour apples or their juice, unripe grapes, pomegranate or sumac syrups, or even dried sour cherries.

The aims of this work were: (1) to review all unpublished or partially published data collected by the authors in Turkey and the Balkans (i.e., in Bosnia and Herzegovina, Serbia, Macedonia, Bulgaria, Albania, as well as in Croatia and Romania, which – despite the fact they have only one portion of their territories located in the Balkans – were considered in their entirety); (2) to review the same plants used for preparing *sarma* from primary folkloric, ethnobotanical, and gastronomic literature from the same countries; and (3) to compare the geographic and cross-cultural diversity of *sarma* in the considered countries.

## Methods

### Field studies

**Figure 1:**
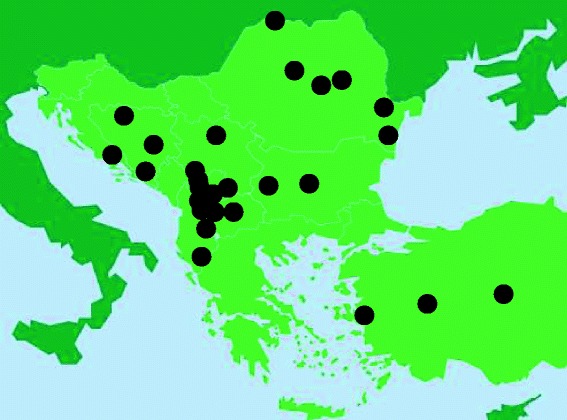
**The study sites.**

Field studies on the use of plants used as wrapping material for *sarma* were conducted during broader ethnobotanical field studies and also via a few *ad hoc* investigations conducted by the authors in the years 2011 to 2014 in the following regions and countries (Figure 1): Dalmatia, Croatia (ŁŁ); Northern and Central Bosnia and Southern Herzegovina (AM, JF, and ŁŁ, respectively); Central and Southern Serbia (ZDS); Kosovo (BM, AH); North, Eastern and Southern Albania (AP); Central and Western Macedonia (SG, AP); Western and Central Bulgaria (AN); Transylvania (CD, NP), Dobruja (AN, YD, AP), Moldavia (AP), and Maramureş regions, Romania (ŁŁ); and in the Aegean and Central Anatolian Turkey (YD).

Informants were asked to mention all plants, whose leaves were used as wrapping material for preparing home-made *sarma*. Ethical guidelines drafted by the International Society of Ethnobiology (http://www.ethnobiology.net) and American Anthropological Association (www.aaanet.org) were rigorously followed and Prior Informed Consent (PIC) was always required before each interview.

Plants were taxonomically identified by the respective researchers and plant nomenclature followed standards set by *The Plant List* [35].

### Literature review

Additionally, published ethnobotanical works, as well as a few folkloric references and gastronomic literature based on field investigations, were considered for the following countries: Albania and Kosovo [12-16,36-39], Bosnia and Herzegovina [5-7,40-43], Bulgaria [8,44-50], Croatia [17-19,51-53], Macedonia [9,10], and Romania [29,54-69]; moreover, for Turkey, ethnobotanical data both published in international and national scholarly journals, as well as those arising from unpublished Master’s and PhD theses were considered [21-26,70-103].

Again, plant nomenclature followed the standards set by *The Plant List* [35].

## Results and discussion

### *Biodiversity of* sarma

Table 1 reports all the plant taxa, whose leaves have been documented to be used as wrapping material for *sarma*. Eighty-seven taxa were found to represent the Turkish and Balkan *sarma* plant heritage, whose knowledge is retained by women. In the list, wild plants were predominant (62%).

**Table 1:** **Plants whose leaves are used for preparing** ***sarma*** **in the studied areas and considered countries**

**Botanical taxon and English common name**	**Botanical family**	**Status**	**Local name(s)**	**Area(s) of use**	**Source(s)**
*Alcea flavovirens* (Boiss. and Buhse.) Iljin Yellow-Green Hollyhock	Malvaceae	W	hero, hiro	Turkey: East Anatolia	[78]
*Alcea hohenackeri* (Boiss. and Huet.) Boiss. Hohenacker’s Hollyhock	Malvaceae	W	fatma gülü, gül hatmi, hero, hiro	Turkey: East Anatolia	[94]
*Alcea kurdica* (Schlecht) Alef Kurdish Hollyhock	Malvaceae	W	hero, heru	Turkey: East Anatolia	[78,95]
*Alcea rosea* L. Common Hollyhock	Malvaceae	W	ружа	Bulgaria: Osogovo Mt.	AN
*Allium ampeloprasum* L*.* Leek	Amaryllidaceae	C	pırasa	Turkey: Izmir	YD
			prasa	Bosnia and Herzegovina: Northern Bosnia	AM
*Allium ursinum* L. Ramsons	Amaryllidaceae	W	левурда	Bulgaria: Lovech area	AN
*Amaranthus viridis* L. Green Amaranth	Amaranthaceae	W	delisirken, hoşguran, kızılca mancar, semlik	Turkey: Şırnak	[76]
*Arctium minus* (Hill) Bernh. Lesser Burdock	Asteraceae	W	dulavratotu, galabah	Turkey: Erzurum	[72]
*Arctium platylepis* (Boiss. & Bal.) Sosn. ex Grossh. Halemhort	Asteraceae	W	baldikeni, deve tabanı	Turkey: NW Anatolia	[103]
*Armoracia rusticana* P.Gaertn., B.Mey & Scherb. Horseradish	Brassicaceae	C	хрян	Bulgaria: Plovdiv area	AN
			hrean, hrin^UK^, torma^HU^	Romania: Dobruja, Transylvania, Maramureș^UK^	AN, AP, YD, CD, ŁŁ
			hren, kren, рeн	Bosnia and Herzegovina and Serbia: diverse areas	AM, JF, ZDS
*Arum conophalloides* Kotschy ex Schott	Araceae	W	yılan bıcağı, yılan yastığı	Turkey: South Anatolia	[77]
*Arum dioscorides* Sm.	Araceae	W	sarmalık, yılan bıçağı, yılan ekmeği, yılan pancarı	Turkey: South and South-Eastern Anatolia	[72,77]
*Arum maculatum* L. Snakeshead	Araceae	W	yılan ekmeği, yılan yastığı	Turkey: West and Central Anatolia	[22]
*Atriplex hortensis* L. Garden Orache	Amaranthaceae	C	градинска лобода	Bulgaria: all over the country	[47], AN
*Atriplex rosea* L. Red Orache	Amaranthaceae	W/C	розова лобода	Bulgaria: all over the country	[8,47]
*Beta trigyna* Waldst. and Kit.	Amaranthaceae	C	mancar, süt mancar,	Turkey: Ankara	[26]
*Beta vulgaris* L. ssp. *vulgaris* convar. *cicla* Beet/Chard	Amaranthaceae	C	pazı	Turkey: Duzce, Turhal, Malatya	[93], YD
			blitva/блитвa	Bosnia Herzegovina and Serbia: diverse areas	AM, ŁŁ, ZDS
*Beta vulgaris* L. ssp. *vulgaris* convar*. vulgaris* var. *altissima* Sugar Beet	Amaranthaceae	C	şekerpancarı, cukorrépa^HU^	Turkey: Afyon Romania: Szekely Land	NP, YD
*Beta vulgaris* L. ssp. *vulgaris* convar*. vulgaris* var. *vulgaris* Beetroot	Amaranthaceae	C	burak^UK^, cékla^HU^	Romania: Moldaviaomania: Szekely Land Romania: Moldavia Maramureș area	[64], ŁŁ
			цвекло	Bulgaria: Bansko, Pirin Mt., Karlovo	[47], AN
			цвeклa	Serbia: diverse areas	ZDS
			pancar	Turkey: Izmir, Malatya	YD
*Brassica oleracea* Acephala group Kale	Brassicaceae	C	kara lahana	Turkey: Black Sea Region, Duzce, Izmit	[81,88,97]
*Brassica oleracea* Capitata Group (both fresh and lacto-fermented [sauerkraut]) Cabbage	Brassicaceae	C	lahana	Turkey: all over the country	YD
			прясно зеле, кисело зеле	Bulgaria: all over the country	[46,47,50], AN
			kupus/купуc	Bosnia Herzegovina, Croatia, and Serbia: all over the country (in Serbia quite exclusively used only lacto-fermented)	AM, JF, ŁŁ, ZDS
			зелка, расол, купус	Macedonia: all over the country	GS
			lakna, liakra	Kosovo and Albania: all over the country	AH, AP
			curechi, káboszta^HU^, káposzta^HU^, varză	Romania: all over the country	CD, NP
*Brassica oleracea* L. var. *gongylodes* Kohlrabi	Brassicaceae	C	алабаш, гулия	Bulgaria: Rhodopes Mt., Dobrostan	[46,47], AN
*Brassica rapa* L. var. *rapa* Turnip	Brassicaceae	C	kırmızı çükündür	Turkey: Düzce	[97]
*Caltha palustris* L. Marsh Marigold	Ranunculaceae	W	bulbuci de baltă, calcea calului	Romania: Moldavia	[59,67]
*Campanula sclerotricha* Boiss. Bellflower	Campanulaceae	W	büyük köklü, çançiçeği, nermedenk	Turkey: Hakkari	[78]
*Centaurea haradjianii* Wagenitz	Asteraceae	W	kaputkulak	Turkey: South Anatolia	[96]
*Cercis siliquastrum* L. Judas Tree	Fabaceae	W/C	Erguvan	Turkey: diverse areas	[103]
*Cirsium arvense* (L.) Scop. Creeping Thistle	Asteraceae	W	köygöçüren, köygöçerten	Turkey: West and Central Anatolia	[22]
*Colocasia esculenta* (L.) Schott Taro	Araceae	C	göleğez	Turkey: Adana, Antalya	YD
*Convolvulus stachydifolius* Choisy	Convolvulaceae	W	sermaşık, sarmaşık	Turkey: Cizre	[76]
*Corylus avellana* L. Hazelnut*	Betulaceae	C	fındık	Turkey: Duzce, Malatya	[97], YD
		W/C	leithi	Kosovo: Pristina area	BM, HA
*Corylus maxima* Mill. Filbert	Betulaceae	C	fındık	Turkey: Duzce, Malatya	[97], YD
*Cydonia oblonga* Mill. Quince	Rosaceae	C	ayva	Turkey: Malatya	YD
			ftoi	Albania: Mt. Korab	[9]
*Heracleum trachyloma* Fisch. & C.A. Mey. Downy cow-parsnip	Apiaceae	W	baldırgan	Turkey: East Anatolia	[103]
*Lactuca sativa* L. Lettuce	Asteraceae	C	marul	Turkey: West Anatolia, Malatya	YD
			маруля	Bulgaria: Sofia area, Plovdiv area	AN
*Malva neglecta* Wallr. Dwarf Mallow	Malvaceae	W	ebegümeci, ebemgümeç, ebemövmeci, tolık, tolk	Turkey: all over the country	[95], YD
*Malva nicaeensis* All. French Mallow	Malvaceae	W	develik, ebegümeci	Turkey: Çanakkale	[75]
*Malva sylvestris* L. Mallow	Malvaceae	W	develik, ebegümeci	Turkey: West Anatolia	[22,75]
*Morus alba* L. White Mulberry	Moraceae	C	akdut, dut, tuye	Turkey: East, West and Central Anatolia	[22,99], YD
*Morus nigra* L. Black Mulberry	Moraceae	C	dut, karadut, tuye	Turkey: all over the country	[22,99], YD
*Morus rubra* L. Red Mulberry	Moraceae	C	mordut, kırmızı dut	Turkey: West and Central Anatolia	[22], YD
*Onopordum illyricum* L. Illyrian Thistle	Asteraceae	W	deli kenger, dolma kenkeri, eşek dikeni	Turkey: Muğla	[101]
*Pelargonium quercetorum* Agnew Turkish Pelargonium	Geraniaceae	W	tolk	Turkey: Hakkari	[78]
*Petasites hybridus* (L.) G*.* Gaertner*,* B*.* Meyer and Scherb. Butterbur	Asteraceae	W	galdirel, kaldırek, kaldirek	Turkey: Manyas	[98]
*Phaseolus vulgaris* L. Bean	Fabaceae	C	fasülye	Turkey: West and East Anatolia, Malatya	[102], YD
			fasole	Romania: Bacau area	AP
			грав^MK^	Albania: Gollobordo^MK^	[13]
			grah, mohune	Bosnia and Herzegovina: Sarajevo area	JF
*Phytolacca americana* L.* Pokeweed	Phytolaccaceae	W	крмус	Macedonia: Strumica area	GS
*Plantago lanceolata* L. Narrowleaf Plantain	Plantaginaceae	W	sinirliot	Turkey: West and Central Anatolia	[22,93]
*Plantago major* L. Broadleaf Plantain	Plantaginaceae	W	belgheviz, damar otu, kesikotu, sinirotu, yara otu	Turkey: East Anatolia, Izmit, Ordu, Samsun,	[72,81,95]
*Primula veris* L. Cowslip	Primulaceae	W	aguliçe, zgjerifet, lulë, lule dashi, lule deshi, lule verdhë, qingji, zgjirifet	Albania: Mt. Korab	[9]
*Primula vulgaris* Huds. Primrose	Primulaceae	W	ak meneksen, çuha çiçeği	Turkey: South Anatolia	[87,77],
*Prunus avium* L. Cherry	Rosaceae	C	kiraz	Turkey: Malatya, Sakarya	[84], YD
*Raphanus raphanistrum* L. Wild Radish	Brassicaceae	W	turpotu	Turkey: West and Central Anatolia, Kahrmanmaras	[22,87]
*Reynoutria japonica* Houtt. Japanese Knotweed	Polygonaceae	W	bambus	Romania: Maramureș	[64], ŁŁ
*Rheum ribes* L. Syrian Rhubarb	Polygonaceae	W	işgın	Turkey: East Anatolia	[103]
*Ribes nigrum* L. Blackcurrent	Grossulariaceae	C	coacăz negru	Romania: Transylvania	[67]
*Rubus idaeus* L. Raspberry	Rosaceae	C	maline	Bosnia and Herzegovina: Sarajevo area	JF
*Rubus caesius* L. Dewberry	Rosaceae	W	капина	Bulgaria: Lovech area	[47]
*Rumex acetosa* L. Sorrel	Polygonaceae	W	ekşi labada, ekşilküçük labada	Turkey: West and Central Anatolia	[22]
			киселец	Bulgaria: Rhodopes Mt. area	[8]
			uthullaçe	Kosovo: Pristina area	BM, AH
			киселицa	Serbia: South and Central regions	ZDS
*Rumex acetosella* L. Red Sorrel	Polygonaceae	W	ebem ekşisi, ekşikulak, kuzukulağı, tırşık	Turkey: East Anatolia	[99]
*Rumex alpinus* L. Alpine Dock	Polygonaceae	W	dağ pazısı, ışgın	Turkey: East Anatolia, Afyon	[72,99]
			ştevia stânelor	Romania: Transylvania	[67]
*Rumex conglomeratus* Murray Sharp Dock	Polygonaceae	W	labada, kuzukulağı, tırşo, tirşik	Turkey: South, East and South-eastern Anatolia, Manyas	[71,76,86,94,98]
*Rumex crispus* L. Curly Dock	Polygonaceae	W	efelek, efelik, kıvırcık labada, tırşo,labada, tirşik	Turkey: West and Central Anatolia, Bursa, Cizre	[22,23,25,72,76,80,84,90], YD
			штавеј	Macedonia: all over the country	GS
			штaвaљ	Serbia: all over the country	ZDS
*Rumex gracilescens* Rech.	Polygonaceae	W	acımancar, efelek, göylek, güyrek	Turkey: Ankara	[26,91]
*Rumex obtusifolius* L. Broad-Leaved Dock	Polygonaceae	W	yabani labada	Turkey: West and Central Anatolia	[22], YD
			ştevie	Romania: Transylvania	[67]
*Rumex olympicus* Boiss.	Polygonaceae	W	ebelek, ilabada	Turkey: Bursa	[72]
*Rumex patientia* L. Patience Dock	Polygonaceae	W	akıllı labada, at kulağı, efelek, evelik, göbede, güylek, labada	Turkey: Thrace, Anatolia	[22-24,80,85,93,98], YD
			лапад	Bulgaria: all over the country	[8,47], AN
			atkulak^TA^, dragomir, măcrisul cucului, ştevie de grădină, ščava^UK^	Romania: Transylvania, Dobruja^TA^, Maramureș^UK^	[64], AN, AP, ŁŁ, YD
			лапад	Bulgaria: all over the country	[8,47], AN
			зeљe	Serbia: diverse areas	ZDS
*Rumex pulcher* L. Fiddle Dock	Polygonaceae	W	labada, ilabada	Turkey: Çanakkale, Izmit	[75,81]
*Rumex tuberosus* L. Swollen Sorrel	Polygonaceae	W	efelek, kuzukıkırdağı	Turkey: East Anatolia, Eskişehir	[70,78,80,95]
*Salvia forskaohlei* L. Forskhal’s Sage	Lamiaceae	W	şalba	Turkey: unspecified Asia Minor	[79]
*Salvia poculata* Náb.	Lamiaceae	W	bareş, öküzpörçüğü, ezmangag	Turkey: East Anatolia	[78,95]
*Salvia sclarea* L. Clary Sage	Lamiaceae	W	tüylü adaçayı, misk adaçayı, pune, ayıkulağı	Turkey: East Anatolia	[99]
*Sinapis arvensis* L. Field Mustard	Brassicaceae	W	hardalotu	Turkey: Tokat	[93]
*Smilax excelsa* L. Smilax	Smilacaceae	W	melevcen	Turkey: unspecified Asia Minor	[79]
*Spinacia oleracea* L. Spinach	Amaranthaceae	C	ıspanak	Turkey: West Anatolia	YD
			спанак	Bulgaria: Sofia area, Plovdiv area	AN
			špinat, španać, cпaнaћ	Bosnia and Herzegovina and Serbia: diverse areas	AM, ZDS
*Solanum tuberosum* L. Potato	Solanaceae	C	компири^MK^	Albania: Gollobordo^MK^	[13], AP
*Silybum marianum* (L.) Gaertn. Milk Thistle	Asteraceae	W	devedikeni	Turkey: diverse areas	[103]
*Symphytum kurdicum* Boiis. and Hausskn. Kurdish Comfrey	Boraginaceae	W	karakafesotu, ezmangag	Turkey: Hakkari	[78]
*Tilia cordata* Miller Small-Leaved Lime	Malvaceae	C/W	blini	Kosovo: Pristina area	BM, AH
*Tilia cordata* Miller and *T. platyphyllos* Scop. Small- and Large-Leaved Lime	Malvaceae	C/W	tei	Romania: diverse areas	[67], AP
*Tilia tomentosa* Moench Silver Lime	Malvaceae	C/W	липа	Bulgaria: Lovech and Tsarevo areas; Turkey: diverse areas	[47,103], AN
*Trachystemon orientalis* (L.) G. Don Abraham-Isaac-Jacob	Boraginaceae	W	galdirik, hodan, ispit, kaldırık, kaldurak otu	Turkey: diverse areas	[83,93,97], YD
*Tussilago farfara* L. Coltsfoot	Asteraceae	W	öksürükotu	Turkey: West and Central Anatolia, Kastamonu	[22,72]
			martilapi^HU^, fehérhátú^HU^, lapu^HU^, podbal, podbielina^PO^ tőltike^HU^	Romania: diverse areas	[60,67-69], AP, CD, ŁŁ, NP
			podbel, podbjel, пoдбeл	Bosnia and Herzegovina and Serbia: diverse areas (in Serbia rarely used)	JF, ZDS
*Urtica dioica* L. Nettle	Urticaceae	W	ısıran	Turkey: South-eastern Anatolia	[74]
			кoпривa	Serbia: diverse areas	ZDS
			hitha, hejtha	Albania: Mt. Korab	[9]
*Vicia faba* L.	Fabaceae	C	бакла	Bulgaria: Karlovo area	[47]
*Vitis labrusca* L. Fox Grape	Vitaceae	C	rrush me erë	Albania: Mt. Korab	[9]
*Vitis sylvestris* Gmelin Wild Grape	Vitaceae	W	çivek, deliasma, lazüzümü	Turkey: Yalova	[100]
*Vitis vinifera* L. Grape	Vitaceae	C	asma, tiri, jur	Turkey: all over the country	[78,83,87,88,90,95], YD
			лоза	Bulgaria: all over the country	[8,46,47,50]
			лоза	Macedonia: all over the country	GS
			rrushi	Kosovo and Albania: all over the countries	AH, AP
			viţă de vie, szőlő^HU^	Romania: diverse areas	[65,67,96], CD, NP
			loza/лoзa	Serbia, Bosnia Herzegovina, and Croatia: diverse areas	AM, JF, ŁŁ, ZDS

C: Cultivated; W: Wild; *: only young/tender leaves; ^HU^folk name recorded among Hungarian minority living in Transylvania, Romania; ^MK^folk name and use recorded among Macedonian minority living in Gollobordo, Albania; ^PO^folk name and use recorded among the Polish minority living in Bukovina, Romania; ^TA^folk name and use recorded (also) among the Tatar minority living in Dobruja, Romania; ^UK^folk name and use recorded (also) among the Ukrainian minority living in the Maramureş area, Romania; data arising from field studies conducted by the authors in the period 2011–2014: AH: Avni Hajdari; AM: Aida Maglajlic; AN: Anely Nedelcheva; AP: Andrea Pieroni; BM: Behxhet Mustafa; CG: Constantin Drăgulescu; GS: Gjoshe Stefkov; JF: Jonathan Ferrier; ŁŁ: Łukasz Łuczaj; NP: Nora Papp; YD: Yunus Dogan; ZDS: Zora Dajić-Stevanović.

Basic ingredients for the stuffing always includes meat or rice, (sautéed) onions, and sometimes, especially in more rural areas, chopped vegetables too (and especially wild vegetables in Moldavia during the spring Orthodox Lent period); in Turkey and Bulgaria *bulgur* (made from the grouts of diverse wheat species), cooked beans as well as *urov* (*Vicia sativa*) can be used in the filling.

In Bulgaria, crushed walnuts may be added to the filling. In Turkish cuisine filling ingredients may include pine kernels (*Pinus pinea*), Black Corinth (*Vitis vinifera*), blackcurrants (*Ribes nigrum*), and even mastic (resin of *Pistacia lentiscus*). An old tradition in Turkey was to also add sour cherries in the filling; however, this tradition is barely alive with only a few traditional restaurants serving the product.

The listed taxa belong to 50 genera and 27 families, with the predominance of Polygonaceae (15%), Malvaceae (11%), Amaranthaceae (11%), Asteraceae (10%), and Brassicaceae (9%). The largest number of taxa was recorded in the genera *Rumex* (11), *Beta* (5), *Alcea* (4), *Brassica* (4), *Malva* (3) and *Arum* (3).

Among them, herbaceous plants represented the majority of the recorded plants (65), while trees (10) and shrubs (2) were mostly from the Rosaceae, Moraceae, Betulaceae and Malvaceae families, while four species were represented by vines.

In Moldavia chopped cabbage and dill branches are often put at the bottom of the pot where *sarma* will be cooked, often adding a pieces of cured pork meat (bacon); in Bulgaria plums are put between the diverse *sarma* units.

While in Moldavia it is customary to add in the cooking pot also home-made *borş* (lacto-fermented wheat bran in water) or unripe grapes (previously cooked in water), in order to provide some sourness (this is not practiced in the case of sauerkraut *sarma*), while the custom to add lemon slices in the pot seems to be prevalent in Turkey and Southern Albania.

**Figure 2:**
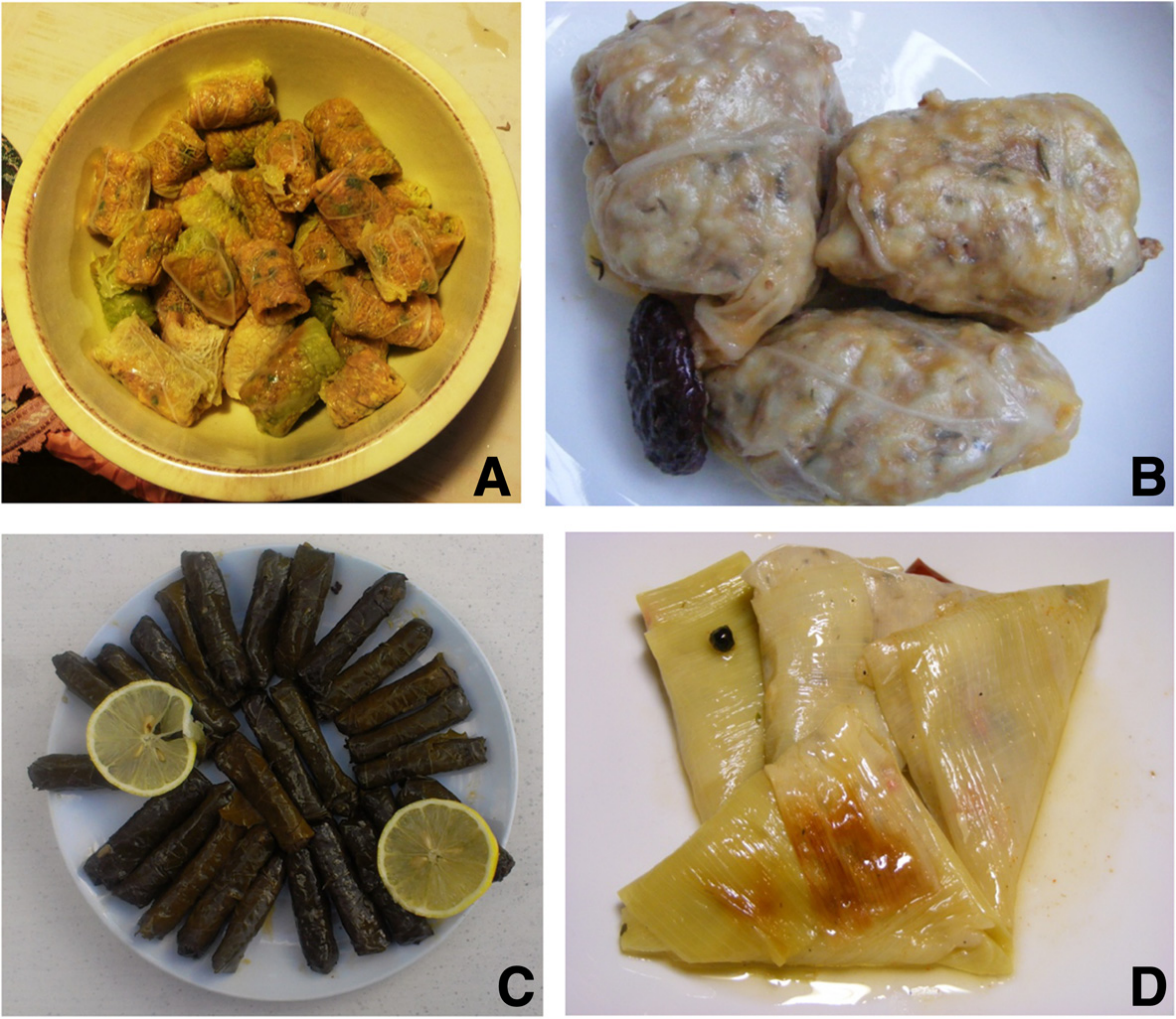
**Diversity of** ***sarma*** **preparations in Turkey and the Balkans; A - Brassica oleracea (just before the cooking process; Romania); B -** ***Brassica oleracea*** **(Bulgaria); C –** ***Vitis vinifera*** **(Turkey)** ***;*** **D –** ***Allium ampeloprasum*** **(Turkey).**

Turkey and Southern Albania *sarma* are typically small and have a cigar-like shape. In Bulgaria *sarma* are larger (Figure 2) and resemble small balls, while in Romania and the other countries may have diverse dimensions. Cigar-like *sarma* are considered appropriate for special guests in the Romanian Moldavia; in this specific case, vine-grape, lime tree, or bean leaves-based cigar-shaped *sarma* are cooked in a group of 6–7 unities, wrapped within larger leaves of cabbage, which are later removed before consuming the *sarma*.

In Moldavia also, a very large cabbage *sarma* exists during the diverse Orthodox Lenten fasting periods. The filling in the Moldavian case is made by a large amount of rice, crashed walnuts, sautéed onions, roughly chopped onions, cabbage, parsnips or carrots, and seasoned with small amounts of ground celery, parsley roots, dill, and whole pepper grains.

*Sarma* prepared from cultivated leek (*Allium ampeloprasum* s.l.) are different from other *sarma* and usually have a triangular shape (Figure 2). This type of *sarma* is part of traditional cuisine found only in some areas of Turkey (e.g. the Aegean region); sometimes *sarma* made by cabbage, kale, dock, and beet can be also prepared in triangular shape.

In general, preparing *sarma* requires special artisanal women expertise and is a time consuming process. Because of the newly introduced lifestyles and maybe changing social role of women, *sarma* is slowly and gradually disappearing from the home cuisine of the studied areas. In Turkey however, *sarma* is still available on the market and a number of women earn money by taking orders at home.

**Figure 3:**
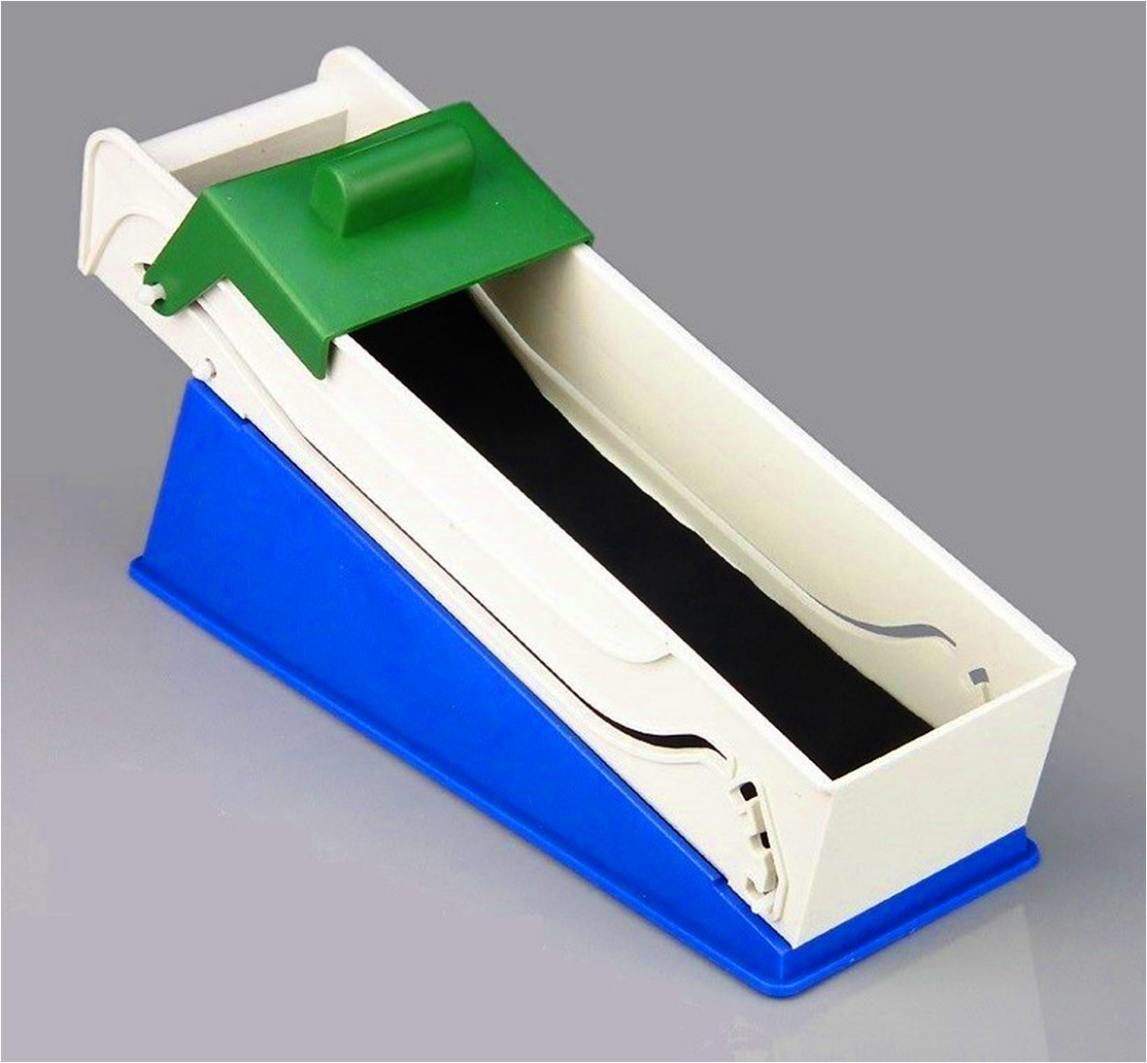
**Turkish “modern” apparatus for making** ***sarma.***

At the same time, it should be noted that in the last decades an apparatus has become commercially available in Turkey that makes it easier to roll the leaves around the filling (esp. grape vine leaves, Figure 3). This tends to produce thin *sarma* having a standardized shape.

**Figure 4:**
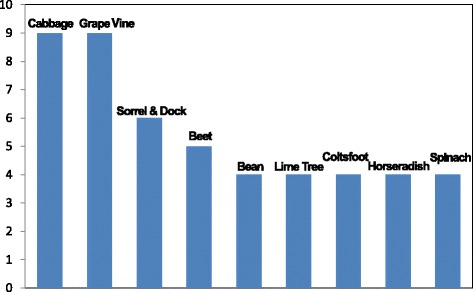
**Most commonly used taxa (number of countries where the use has been recorded).**

Figure 4 shows the most widely used *sarma* leaves in the considered countries. Cabbage and grape vine, and to a minor extent, beet, dock and sorrel, lime tree, spinach, beans, and horseradish are plants that have been reported to have been used in at least four countries.

### *Botany and sensory characteristics of* sarma’s *leaves*

According to our sources, three principal criteria guide the rationale behind the use of plant leaves for *sarma*: (1) leaves have to be large enough (ideally the size of one’s palm) to wrap what is considered to be a proper amount of the filling. *Sarma* is generally eaten in one or two bites, or, even more as in the case of the large cabbage *sarma* prepared during the Orthodox Lent in Moldavia; (2) leaves must be strong enough to not degenerate during the cooking process while retaining the flavour of the filling; and (3) leaves may add a specific texture (e.g., bean and grape vine leaves) or taste to the filling (e.g., lime tree leaves, cabbage, horseradish, coltsfoot, leek). The aforementioned requirements of leaves used for *sarma*, can be linked with some botanical-morphological and, or phytochemical characteristics.

Concerning the first criterion, which dictates the general rule of “one leaf lamina – one *sarma*”, we have recorded only some exceptions among cultivated leafy vegetables: cabbage outer leaves are sometimes divided into two or three parts, which individually wrapped around the filling; the same may be (more rarely) done with leaves of spinach, beets, horseradish, or lettuce.

Leaf shape is variable but mostly consist of round (*Brassica oleracea*), reniform/kidney (*Tussilago farfara*), ovate (*Corylus avellana*), cordate (*Smilax excelsa, Tilia tomentosa*), elliptic (*Allium ursinum*), or lanceolate (*Armoracia rusticana*, *Rumex* spp., *Arum* spp.) shapes, including various intermediate forms.

The dominant leaf types make it possible to wrap cigar-shaped *sarma*, while for a few species rosette (*Brassica oleracea*, *Cirsium arvense, Primula* spp., *Plantago* spp.), basal (*Rumex* spp., *Arum* spp.) and even stem leaves (*Alcea* spp., *Malva* spp., *Corylus avellana*, *Cydonia oblonga*) are used.

Interestingly, when using leaves in which the lower (or both) surfaces are covered with trichomes (with varying densities) (e.g., *Tussilago farfara*, *Tilia tomentosa*, *Salvia* spp., *Petasites hybridus*), to avoid their unpleasant effect, only young leaves are normally collected; this approach also allows avoidance of the thorns of *Cirsium arvense* leaves and the glandular trichomes of *Pelargonium quercetorum.* Some of the species (*Morus* and *Vitis* spp.) have well expressed heterophylly and thus, to identify the most suitable leaves, requires specific knowledge of the morphology and ecological plasticity of the species.

Regardless of the morphological characteristics of the leaves, most leaf types go through a preliminary heat treatment before being used as wrapping material, thus increasing their flexibility.

Some leaves (esp. cabbage, grape vine, and lime tree) are also preserved via lacto-fermentation to ensure their availability during winter.

### Most uncommon reports

**Figure 5:**
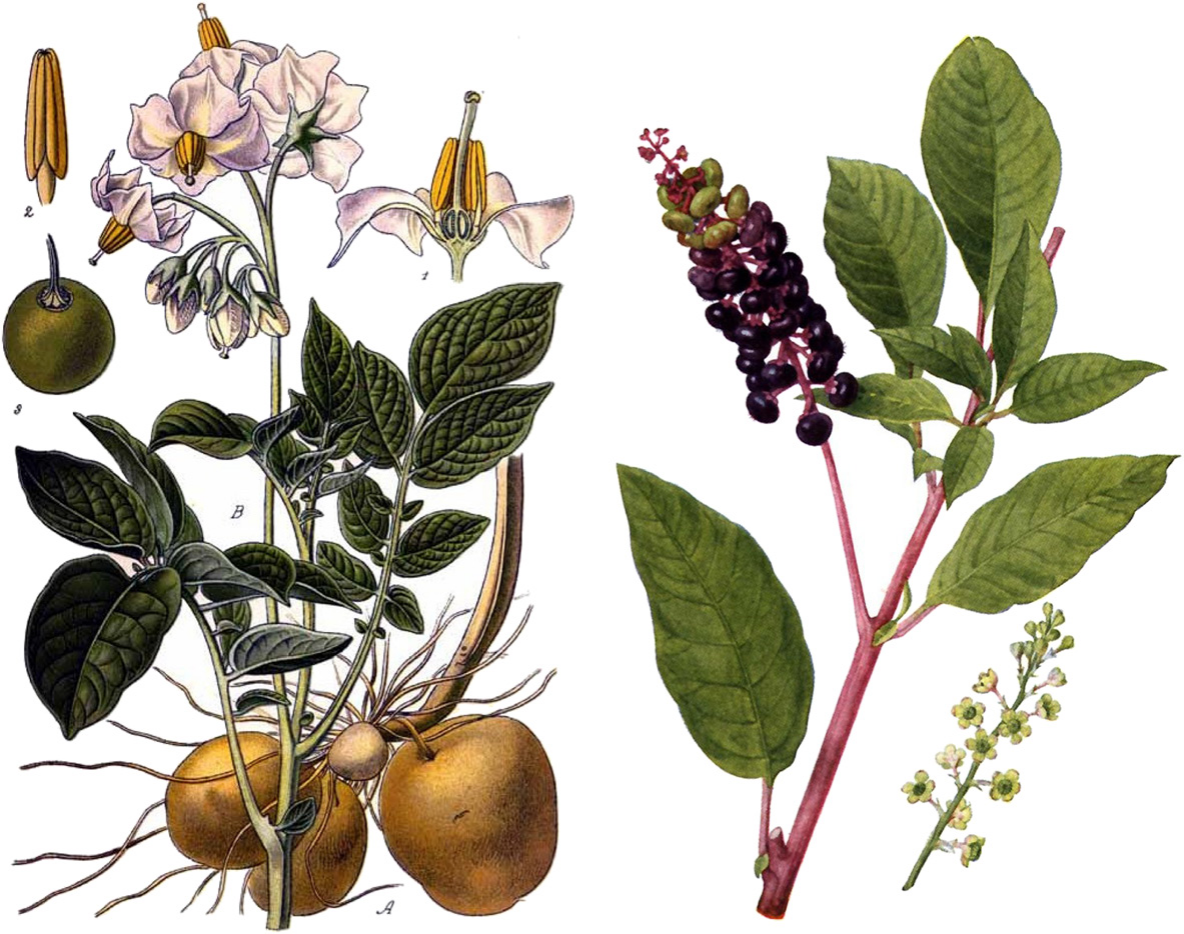
**Unusual food ingredients used for preparing** ***sarma*** **: young potato and pokeberry (** ***Phytolacca americana*** **) leaves.**

Apart from a few endemic species (*Centaurea haradjianii, Rumex gracilescens* and *R. olympicus* in Turkey), our findings also reveal the use in *sarma* of leaves that are uncommonly used as food items. A few of these are considered lightly toxic ingredients, such us potato leaves in North-Eastern Albania, leaves of *Arum, Convolvulus,* and *Smilax* species in Turkey, of *Phytolacca americana* in Macedonia (Figure 5)*,* and of *Tussilago farfara* in Turkey and Romania.

Toxicity is removed via preliminary blanching of the leaves of *Arum conophalloides, Arum dioscorides, Arum maculatum, Colocasia esculenta, Caltha palustris,* and *Smilax excelsa.*

The consumption of these taxa could be linked to their broad leaf shape, large ecological, and seasonal availability in specific areas, but also to specific sensory characteristics, which in Southern Europe are also sometimes linked to perceived medicinal values [104].

It is possible to categorize the recorded leaves accordingly to their taste after cooking: a few (e.g., *Allium* spp.) may provide garlic- and leek-like tastes; others (e.g., *Rumex*, *Corylus, Cydonia, Morus, Tilia, Vitis* spp.) have a sour or a light astringent taste; or may provide bitter taste (e.g., potato leaves, *Arctium, Centaurea, Cirsium, Petasites, Tussilago,* and *Lactuca* spp.); a few provide cabbage-like (e.g., horseradish leaves, *Caltha palustris*), aromatic (*Salvia sclarea*), or even pungent tastes (*Arum* spp.). All of the aforementioned species are able to add a specific flavour to the final *sarma* taste and contribute in this way to an important diversification of this traditional elements of the festivity diets.

However, an important portion of the quoted leaves have a neutral taste (spinach, beans, beet, lime tree leaves), sometimes coupled with mucilaginous characteristics (e.g., *Alcea* and *Malva* spp.).

### Cross-cultural comparison

**Figure 6:**
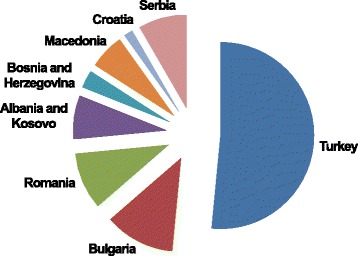
**Biodiversity of** ***sarma*** **in the considered countries (according to the number of recorded plant taxa).**

Figure 6 shows the distribution of the plant biodiversity of *sarma* among the considered countries. Turkey has the greatest diversity of *sarma* leaves (n = 68 taxa, representing 78% of the overall recorded plants), while the Balkan countries listed significantly fewer plants, with a richer *sarma* diversity in Bulgaria (n = 16) and Romania (n = 14). The biodiversity of the *sarma* leaves tends to significantly decrease towards the Mediterranean cultural area (Dalmatia/Croatia) reflecting former borders of the Ottoman Empire. Only a small number of plants (16%) were reported for more than one country (12). These are mainly cultivated edible greens (e.g., *Armoracia rusticana, Beta vulgaris*, *Brassica oleracea*, *Lactuca sativa, Spinacia oleracea* and their cultivars), with legumes (e.g., *Phaseolus vulgaris*), fruit trees and shrubs (e.g., *Cydonia oblonga, Vitis* spp.).

Among the quoted wild plants, dock and sorrel (*Rumex* spp.) predominate; however, *Rumex* spp. leaves are widely used also in other preparations in the local diets of Turkey and Balkan mountainous pastoralist communities [1] and represent important food items for populations that originated in Central Asia, as demonstrated by a recent study among the Tatars of Romania [61].

Most of the quoted wild plants are, however, well-known in the studied areas as edible plants, and are used for preparing salads, soups, and pies [1,7,8,21,22].

According to Table 1, the greatest diversity of *sarma* types are consumed within Turkey, between Western Anatolia (and Izmir and its surroundings, e.g., *Allium ampeloprasum, Beta vulgaris, Lactuca sativa, Morus rubra, Phaseolus vulgaris, Rumex obtusifolius,* and *Spinacia oleracea*) and Eastern Anatolia, especially Malatya and its surroundings (e.g., *Beta vulgaris, Cydonia oblonga, Phaseolus vulgaris,* and *Lactuca sativa*).

### *The dynamism of* sarma*’s cultural meanings*

*Sarma* leaves retain diverse cultural- and place-specific meanings for each of the studied areas, and these meanings have changed, and are most probably continuing to change, over time.

While the cultural meaning of *sarma* in the Ottoman (and then mainly Islamic) cuisines is indisputable, *sarma* seems to be also strongly related to traditional Orthodox festivity meals, especially in Bulgaria and Romania (e.g., Christmas Eve, All Souls’ Day, and especially Easter) but also among the Roman-Catholic Croats.

For example, on Christmas Eve in Bulgaria and in the whole Orthodox Lent periods in Romania, vegetarian *sarma* represent the main dish.

Moreover, in the Bulgarian folk customs, the grape vine was mainly considered as the starting material (fruits) for producing wine; during the Communist period however, many Bulgarian workers moved to Northern African (Arabic) countries (e.g., Libya, Algeria), where they learned to prepare *sarma* from grape leaves where *sarma* is considered a typical Arabic meal (and also commonly used in Greece). Grape vine-based *sarma* became popular during that time and cabbage and grape leaves now represent the most commonly used *sarma* leaves of the Bulgarian cuisine.

Whereas the first cookbook written in the Bulgarian language (printed in 1870 in Istanbul [49]) included some *sarma* recipes in which hazelnut leaves were used and vine branches were placed at the bottom of the pot. Neither of these gastronomic uses were found in our field studies, nor in the primary folkloric sources of the twentieth century.

**Figure 7:**
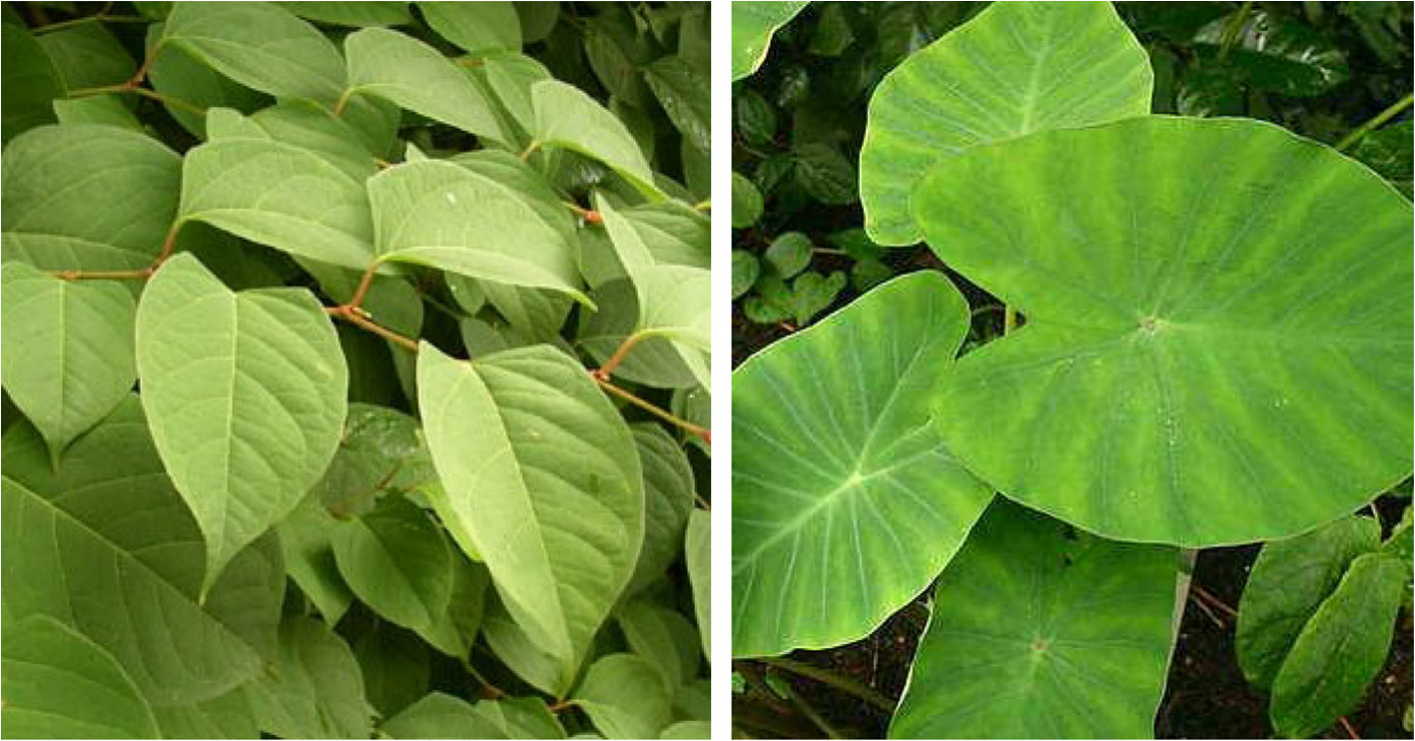
**Novel** ***sarma*** **ingredients:** ***Reynoutria japonica*** **and** ***Colocasia esculenta.***

Finally, novel *sarma* plants, such as *Reynoutria japonica* in North-Western Romania, *Colocasia esculenta* in Turkey (Figure 7), and *Phytolacca americana* in Macedonia demonstrate the dynamic nature of folk cuisines. Within certain eco-zones, these introduced plants have probably represented the most widely available plant resources, which may have led local populations to experiment with new ingredients in their cuisines. These novelties may have in turn diffused via cultural exchange into neighboring areas.

## Conclusion

The findings of this study show a remarkable diversity of *sarma* preparations across the considered countries, stemming from an unexpectedly diverse selection of wild plant leaves.

Turkey retains approximately half of the entire *sarma* plant biodiversity recorded in the considered countries, thus confirming the strong link between this culinary preparation and the Ottoman cuisine of the last four centuries.

However, the cultural meanings of *sarma* also in the Balkan Orthodox customs, and notably in Bulgaria, Romania, and Serbia, is remarkable, and demonstrates the extremely dynamic and changeable nature of folk ethnobotanical practices.

The rationale behind the choice of the appropriate leaves for *sarma* include shape, size, texture, and the ecological and seasonal availability of specific plant leaves; moreover, the importance of taste (and possibly perceived medicinal values) in the choice of the most appropriate leaf wrap was evidenced. This medicinal evidence may also account for the use of lightly toxic plants; however, the human ecological significance of the consumption of these leaves should be clarified case-by-case, and by analyzing the specific historical, anthropological, and environmental contexts. For example, regarding the consumption of potato leaves (sometimes as *sarma* wrapping material, but also in other food contexts) that we recorded in a few villages in North-Eastern Albania and on the Macedonian side of Korab Mountain [9,13], we propose that this might be the result of an extreme environmental adaptation by the local populations after the introduction of the potato crop (around the end of the nineteenth century). The subsequent demographic pressures may have forced locals to permanently inhabit inhospitable summer pastures, where the availability of edible greens in the first spring months (due to the severe winter climatic conditions) could have been extremely limited.

We believe that this rich ethnobiological heritage may be of interest to scholars and folkloric museums, and especially useful for re-evaluating local food niche markets and avant-garde gastronomic trends [105]. In fact, both of these trajectories are increasingly focused on reconsolidating the healthy and sustainable foods practices of folk cuisines, which are often linked with the ‘sense-of-place’ of a given biocultural *oikos* (a.k.a., *terroir*). At the same time, the valorization of reservoirs of ethnobotanical knowledge could have a tremendous impact upon the food sovereignty and health strategies of rural communities in South-Eastern Europe [106].
